# Characterization of a *Diaporthe toxica* Strain: Growth, Spore Formation, Phomopsin-A, and Alkaloids Production on Lupins

**DOI:** 10.3390/toxins16110481

**Published:** 2024-11-07

**Authors:** Francesco Buccioni, Chiara Rossi, Annalisa Serio, Federico Fanti, Antonello Paparella

**Affiliations:** Department of Bioscience and Technology for Food, Agriculture and Environment, University of Teramo, Via R. Balzarini 1, 64100 Teramo, Italy; fbuccioni@unite.it (F.B.); aserio@unite.it (A.S.); ffanti@unite.it (F.F.); apaparella@unite.it (A.P.)

**Keywords:** lupin, *Diaporthe toxica*, endophytic fungi, mycotoxins, phomopsin-A, alkaloids

## Abstract

The growing interest in vegetable proteins, namely those derived from lupins, has raised concerns over potential safety risks associated with these food products. Lupin serves as the main host for the mycotoxin-producing fungus called *Diaporthe toxica*. This species, which is associated with animal diseases, has been scarcely characterized. Recently, phomopsin-A (PHO-A), the main mycotoxin produced by *D. toxica*, was found to be harmful to humans. Therefore, this study aimed at characterizing *D. toxica* growth and spore formation both in vitro and on lupin samples. In addition, the production of PHO-A and alkaloids was investigated on lupin beans by using three different inoculation methods. Particularly, growth and spore production were evaluated on different media, while PHO-A and alkaloid production were determined by means of µSPE extraction followed by UHPLC-MS/MS and HPLC-MS/MS, respectively. The results have demonstrated differences in growth on different media, with potato and oat-flakes-based media being the best options. Conversely, *D. toxica* was not able to produce spores on agar media, but only on lupin beans. Moreover, a thorough analysis of PHO-A production revealed an increase over time, reaching values up to 1082.17 ppm after 21 days on artificially rehydrated samples. On the other side, the analysis of alkaloids revealed impressive results, as this species produced great quantities of the quinolizidine alkaloids (QA) that are normally present in lupin seeds such as lupanine, sparteine, multiflorine, and hydroxylupanine. On balance, considering these results, different metabolic pathways were demonstrated in *D. toxica*, which are not adequately described in the existing literature. These data are of paramount importance to deepen the knowledge about a fungal species that is important to ensure the safety of lupin and lupin-based products.

## 1. Introduction

Filamentous fungi have always been related to a low risk for humans. However, many of them are able to produce mycotoxins which can be extremely harmful to humans.

Mycotoxins are low-molecular-weight secondary metabolites of filamentous fungi that can cause disease in both humans and animals. Their effect on the human body depends on the type of mycotoxin, the level of exposure, and the intrinsic characteristics of the patient. Therefore, their identification and management are of utmost importance for maintaining a high level of safety [1]. Even if many mycotoxins, such as aflatoxins and ochratoxins, have already been identified and classified for their toxicity, others are still under evaluation by the scientific community. In fact, this class of compounds exhibits a wide range of chemical structures and significant variations in their physical, chemical, and biological properties [2,3]. Phomopsins (PHOs) are mycotoxins produced by the fungus *Diaporthe toxica*. *D. toxica* is the teleomorphic stage in the life cycle of the toxigenic fungal species *Phomopsis leptostromiformis*, a phytopathogenic, endophyte, and saprobe fungus that has *Lupinus* spp. as its main host. It has been observed in several countries for causing stem and pod blight in the major lupin species. In addition, in the saprophytic stage, the infection of pods and lupin seeds, along with toxins synthesis, is facilitated by humidity [4,5,6]. *D. toxica* recently gained the attention of the scientific community due to its ability to produce PHOs, particularly PHO-A [7]. PHO-A has been identified to be harmful to grazing animals, causing a disease called “lupinosis” with liver damage as the main lesion in sheep and occasionally cattle, horses, pigs, and goats [8]. Furthermore, lupin beans contain quinolizidine alkaloids (QA), a class of anti-nutritional compounds that includes more than 150 different compounds having a wide range of implications on human health [9].

Recently, together with the increased consumption of lupins, concerns about the potential toxicity of PHOs for humans have been raised, leading to the publication of a scientific opinion by the European Food Safety Authority (EFSA) [10]. Although the pathogenesis of lupinosis has been thoroughly described, only a few studies characterized *D. toxica* and its cultivation techniques, as well as its reproduction strategies and defensive mechanisms. Moreover, there is currently no scientific evidence about the production of alkaloids in lupin beans, despite it being a common feature in many endophytic fungi. Therefore, the aim of this study is to characterize *D. toxica* with respect to its cultivation in different media, considering also the formation of spores, mycotoxins, and other secondary metabolites production, and combining several approaches, including an in situ evaluation of its metabolism on lupin beans.

## 2. Results

### 2.1. Fungal Mycelium Growth and Spore Production

**Figure 1:**
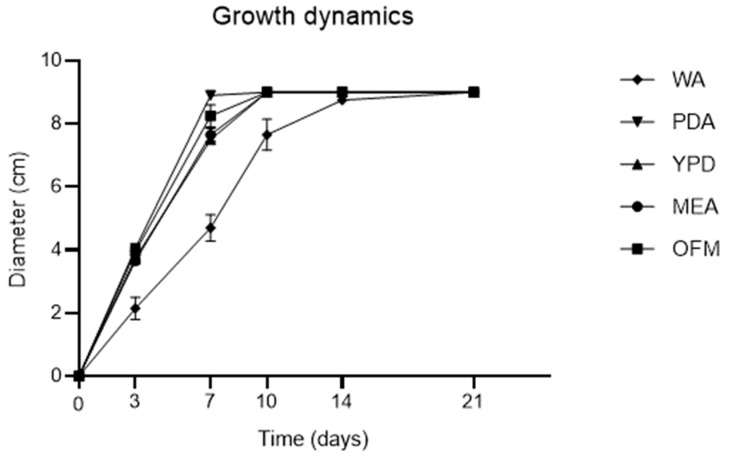
Mycelium growth of *D. toxica* DSM 1894 on different agar media, after 0, 3, 7, 10, 14, and 21 days of incubation at 25.0 ± 0.1 °C. WA: Water Agar; PDA: Potato Dextrose Agar; YPD: Yeast, Peptone, Dextrose Agar; MEA: Malt Extract Agar; OFM: Oat Flake Medium. Values in the figure represent the mean of three replicates ± standard deviation.

**Figure 2:**
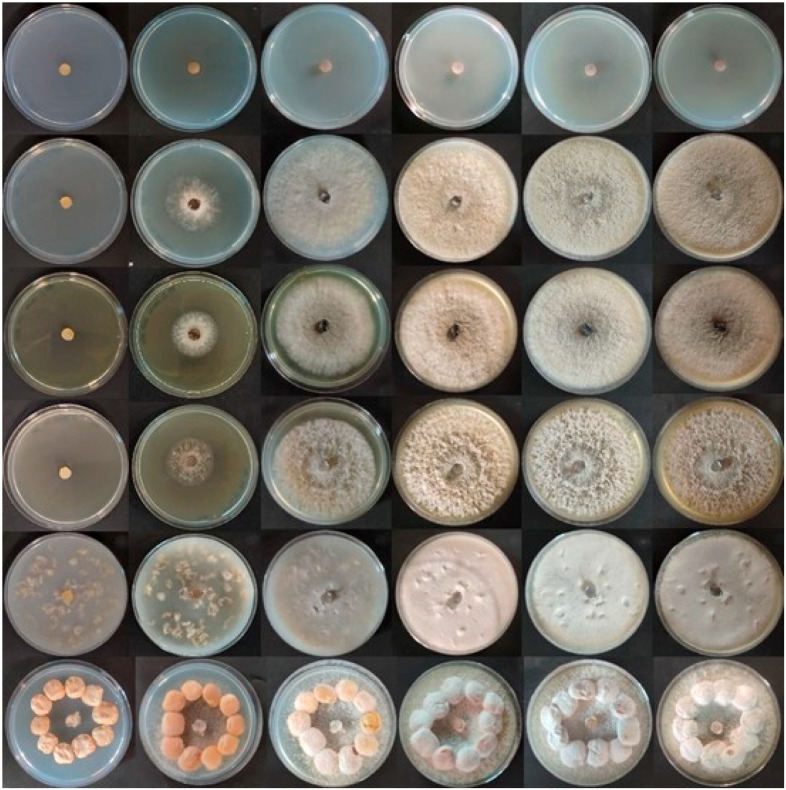
Observation of *D. toxica* DSM 1894 growth over time. From top to bottom: Water Agar (WA), Potato Dextrose Agar (PDA), Yeast, Peptone, Dextrose Agar (YPD), Malt Extract Agar (MEA), Oat Flake Medium (OFM), WA with lupins. From left to right: 0, 3, 7, 10, 14, and 21 days of incubation at 25.0 ± 0.1 °C.

Fungal growth dynamics were evaluated on different substrates, after progressive incubation times at 25 °C, by measuring hyphae diameters. As it can be observed in Figure 1 and Figure 2, Potato Dextrose Agar (PDA) and Oat Flake Medium (OFM) substrates accelerated the growth of the mycelium, which reached the maximum diameter of 90 mm after 10 days of incubation. The growth on Malt Extract Agar (MEA) and Yeast, Peptone, Dextrose Agar (YPD) was slightly slower, with a similar rate. Instead, the mycelium on Water Agar (WA) was significantly slower and reached its maximum expansion only after 21 days of incubation. Moreover, the hyphae on WA were nearly transparent (Figure 2).

Spore production was evaluated on the different media by means of optical microscopy. Results evidenced the presence of reproductive ascospores only on WA with lupins, which is different from previous studies performed on the same microorganism [11].

### 2.2. PHO-A Production

**Figure 3:**
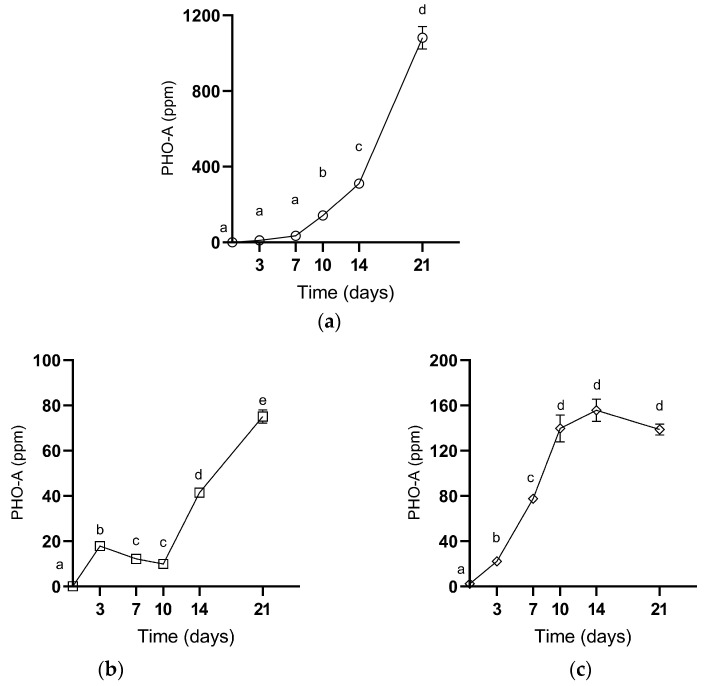
PHO-A production on (**a**) RHC (Rehydrated, central inoculum), (**b**) AHC (Commercial, central inoculum), (**c**) AHL (Commercial, inoculum on the seed) lupin samples inoculated with *D. toxica* DSM 1894. Values in the figures represent the mean of three replicates ± standard deviation. Different lowercase letters above samples represent significant differences (*p* < 0.05) among samples.

In the samples named RHC (Rehydrated Central inoculum), AHC (Already Hydrated, Central inoculum), and AHL (Already Hydrated, inoculum on Lupin), following the different inoculation procedures, PHO-A production was observed over time (Figure 3). In detail, the RHC samples of PHO-A followed a regular pattern, increasing from 0.17 ppm at the beginning of the incubation to 34.23 ppm on day seven. Successively, it significantly increased to 141.17 ppm at day 10 and up to 310.18 at day 14. At the end of the incubation, RHC showed the highest value among all the samples, with a concentration of 1082.17 ppm. On the other side, in both the AHC and AHL samples, the PHO-A concentration remained lower, reaching a maximum of 75.09 ppm and 155.83 ppm, respectively. Notably, PHO-A had an exponential increase in the RHC samples, whereas it followed different pathways in the AHC and AHL samples. The mycotoxin content in the control samples always remained lower than the LOD.

### 2.3. QA Variation in Lupin Samples

**Figure 4:**
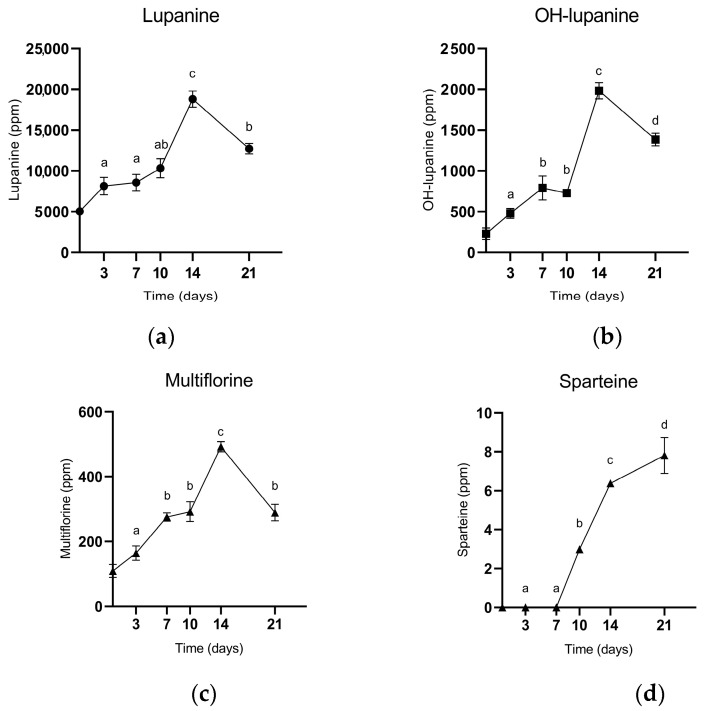
Evolution of (**a**) lupanine, (**b**) hydroxylupanine, (**c**) multiflorine, and (**d**) sparteine production over time. Values in the figures represent the mean of three replicates ± standard deviation. Different lowercase letters above samples represent significant differences (*p* < 0.05) among samples.

The analysis of the lupin bean samples revealed a particular behaviour of *D. toxica* during its growth. Lupanine, sparteine, multiflorine, and hydroxylupanine significantly increased over time until day 14 of incubation (Figure 4). In particular, lupanine at its highest value was almost four times the concentration at the beginning, starting from 5041.00 ± 169.56 ppm and significantly increasing up to 18,796.67 ± 996.31 ppm. The increase in multiflorine and hydroxylupanine was even more evident, starting from 109.42 ± 20.19 and 230.67 ± 71.99 and reaching values up to 492.23 ± 15.67 and 492.23 ± 101.28. Vice versa, sparteine was the only QA that did not sensibly change. Notably, the alkaloid content in the non-inoculated control samples was almost constant.

## 3. Discussion

Growth data demonstrate a rapid adaptation of *D. toxica* DSM 1894 in almost every media, especially in MEA and PDA. This feature meets the expectations since species belonging to the genus *Phomopsis* have been frequently isolated from both potatoes and oats [12,13]. In fact, as *D. toxica* and its anamorph *Phomopsis* are endophytes symbiotically related to their host plants, a rapid hyphal development on plant-based media is reasonable [14]. On the other hand, YPD and MEA contain all the basic requirements for fungal growth, so that *D. toxica* can easily adapt and grow. This behaviour agrees with the research carried out by Crowther et al. (2018) on the differences between natural and artificial media in fungal growth. In fact, although synthetic media can be useful in capturing some aspects of fungal metabolism, they inexorably fail to reproduce the chemical heterogeneity of natural resources vital for fungal development. For the same reason, considering the growth on WA, the low growth rate is likely to be due to the scarcity of nutrients in the medium [15].

Spore production was only achieved on WA with lupins, in contrast with what was observed in other studies, in which different authors agreed on the idea that low-nutrient media and low growth rate enhance spore production in *D. toxica*, and therefore indicating WA as the best medium for sporulation [11,15]. Instead, based on the results presented in this study, lupin bean is the most favourable substrate for *D. toxica* growth and reproduction, as reported in other studies, where lupin, specifically *L. angustifolius*, serves as a significant growth medium for this microorganism [16]. In fact, while *Phomopsis* spp. produces black pycnidial stromata, which can contain alpha- and beta-conidia, its teleomorphic stage *Diaporthe toxica* has been demonstrated to produce ascomata containing ellipsoidal ascospores, according to the information reported by Udayanga et al. (2011) [17]. These spores were observed only on WA with lupin beans. Nonetheless, in this study, the fungus was not able to produce a significant number of spores, in agreement with other studies that describe how challenging is to replicate sporulation of *D. toxica* in controlled conditions. In fact, up to 52 days are needed to produce spores on agar media, and even more months on lupin beans, depending on the season [18,19].

Concerning the PHO-A production, different behaviours were observed in the three different scenarios described in the experimental plan. In general, the PHO-A production reached higher levels compared to the concentrations reported in other studies. In fact, in the research conducted by Kunz et al. (2021) on PHO-A produced by *D. toxica* DSM 1894 on peas, PHO-A was below the limit of detection (LOD) until day three of incubation, while in the present work it reached 10.84 ± 0.31 ppm at the same time. Similar values were reached on peas only after day 14, where the PHO-A content ranged from 4.49 to 34.3 ppm [20]. As observed by Kunz et al. (2021) on peas, we found a constant increase in mycotoxin production over time. On the other side, in the AHC and AHL samples, the PHO-A concentration remained lower, reaching a maximum of 75.09 ppm and 155.83 ppm, respectively. The high concentration observed in the RHC samples confirmed the expectations, as the a_w_ value of 0.98 ± 0.01 is the best condition for growth and mycotoxin production, considering also that lupin is likely a better host than peas for the fungus features [20]. However, the UHPLC-MS/MS analysis of the AHC and AHL samples at different incubation times revealed an increase in PHO-A, suggesting a potential increase in PHO-A in ready-to-eat lupins when the raw material is heavily contaminated and not properly managed at industrial level.

A very important characteristic explained by the results presented in this study was the production of QA during fungal growth on lupin beans. QA are fundamental molecules in the growth, survival, and resistance of the lupin plant whose production has been frequently encountered in various endophytic fungi on other substrates, such as those belonging to the *Claviceps* genus that produces ergot alkaloids, or by *Aspergillus fumigatus* that produces asperfumin [21,22]. The mechanism behind the production of alkaloids by microorganisms is still unknown, but it has been demonstrated that endophytes can produce secondary metabolites mostly equivalent to their hosts. Particularly, *Phomopsis* spp. can produce a wide variety of compounds on different plants [23]. Considering the available literature, it was demonstrated that both *Phomopsis* spp. and its teleomorph *Diaporthe* can produce alkaloids with various important biological activities, but to the best of our knowledge, no evidence exists about the species *D. toxica* and the production of QA on lupin beans [13,20,22,23]. Even if this symbiotic relationship confers the enhanced defence of the host plant, it could be particularly hazardous when lupins are destined for food or feed products. In fact, not only PHO-A can be harmful to humans and animals, but also QA in high concentration can cause severe illness by acting as the acetylcholine receptor agonist, decreasing coronary flow and heart rate [24,25,26,27]. For this reason, based on our data, the inhibition of *D. toxica* becomes even more important and greatly relevant to keeping the QA content at a reasonable and safe concentration. In the present study, in most cases after 14 days of incubation, alkaloid concentrations decreased. In this respect, it could be hypothesized that *D. toxica* ceases producing alkaloids due to the lack of nutrients in the growth media, including lupin beans. In agreement with Helander et al. (2016), alkaloid production by endophytes is strongly related to nutrient availability in soil due to their nitrogen-based synthesis [28]. Consequently, not only can *D. toxica* not produce alkaloids after 14 days, but it possibly starts degrading the already present ones as new carbon sources for its basic metabolism. This behaviour is not completely new, although has never been observed in *D. toxica*. In fact, some studies demonstrated the ability of certain bacteria, such as *Xanthomonas oryzae*, to reduce lupanine concentrations up to 85%, and of some fungi, such as *Aspergillus oryzae* and *Saccharomices cerevisiae*, to convert other classes of alkaloids into other low-toxicity or even useful compounds [29,30]. In the present study, lupanine, multiflorine, and hydroxylupanine were reduced by roughly half after day 14 of incubation, while sparteine concentration continued to increase up to 7.81 ± 0.01 ppm. Consequently, this aspect must also be evaluated since not every alkaloid undergoes degradation at the same time or at the same rate.

## 4. Conclusions

The results showed a great adaptation of *D. toxica* to different growth media, either synthetic or natural, with a preference for those containing natural substrates that usually host this fungus. Some criticalities related to spore production emerged. However, PHO-A production was impressive on lupin beans, where the fungus was also able to produce the same alkaloids used by the plant as a defence during growth. Different testable hypotheses for future studies arise from the present study. However, a deeper understanding of spore production is needed, as well as an investigation of the alkaloid synthesis pathway by *D. toxica*. Finally, this study contributes to expanding the knowledge of the physiology and metabolism of *D. toxica*. Moreover, the scientific evidence provided by this research is useful for identifying possible strategies to reduce infections from *D. toxica* in lupin, as well as PHO-A and alkaloid production.

## 5. Materials and Methods

### 5.1. Fungal Strain

The toxigenic strain *Diaporthe toxica* DSM 1894 was purchased from Leibniz Institute (DSMZ-German Collection of Microorganisms and Cell Cultures GmbH, Braunschweig, Germany) and used for the analyses carried out in this study. Following the company’s guidelines, the strain was routinely cultivated on Oat Flake Medium (OFM) and prepared as follows: 30 g natural oat flakes were boiled under agitation for 10 min. Successively, 20 g technical agar was added, and the volume was filled up to 1 L. Finally, the medium was sterilized at 121 °C for 15 min and shaken before pouring it into sterile Petri dishes.

### 5.2. Fungal Mycelium Growth and Spore Production

#### 5.2.1. Media Preparation and Inoculation Procedure

To evaluate the mycelium growth and the spore production, different agar media were prepared as follows: OFM was prepared as previously described in Section 5.1. Potato Dextrose Agar (PDA) was prepared as follows: 200 g of potatoes were peeled and diced uniformly, then boiled for 20 min in 800 mL distilled water under agitation. The extract was filtrated by means of a sterile gauze, collected in a flask, and added with 20 g agar. Contemporarily, 20 g of dextrose was dissolved into 200 mL of distilled water. The solutions were sterilized and successively mixed and then plated into sterile Petri dishes. Commercially available Yeast, Peptone, Dextrose agar (YPD) and Malt Extract Agar (MEA) were prepared according to the instructions given by the company (Liofilchem S.r.l., Roseto degli Abruzzi (Italy)). Water agar was prepared as a substrate capable of enhancing spore formation by dissolving 18 g/L of agar in distilled water, and then sterilized and plated into sterile Petri dishes [14]. Spore production was also evaluated on WA with dry lupins placed in a circle onto the agar surface and hydrated for 30 min with sterile tap water in a 1:2 *w/v* ratio, until a_w_ reached 0.98, which is described in previous studies as the best growth and PHO production condition for *D. toxica* [20]. The described agar media were inoculated by picking up a round, an 8 mm diameter hyphal portion from a fully grown fungus (5 days), cultivated on OFM agar, and placing the plug in the centre of the agar plates. Tests were performed in triplicate.

#### 5.2.2. Fungal Mycelium Growth and Spore Determination

Fungal growth analysis on the different substrates, reported in paragraph 5.2.1, was evaluated by means of diameter growth measurements. The mycelium diameter was measured using a calliper immediately after inoculation and after 3, 7, 10, 14, and 21 days of incubation at 25.0 ± 0.1 °C. Tests were performed in triplicate.

The evaluation of the spore production was performed by collecting spores with a sterilized physiological solution from the 7 days-grown fungus on each different agar media, according to Molina-Hernandez et al. (2022) [31]. Microscope slides were subsequently prepared with 10 μL of spore suspension and observed by means of an Olympus BX60 optical microscope (Olympus, Tokio, Japan), coupled with a JVC TK-C1380 Colour Video Camera (JVC, Yokohama, Japan).

### 5.3. PHO-A and Alkaloids Determination

#### 5.3.1. Media Preparation and Inoculation Procedure

**Figure 5:**
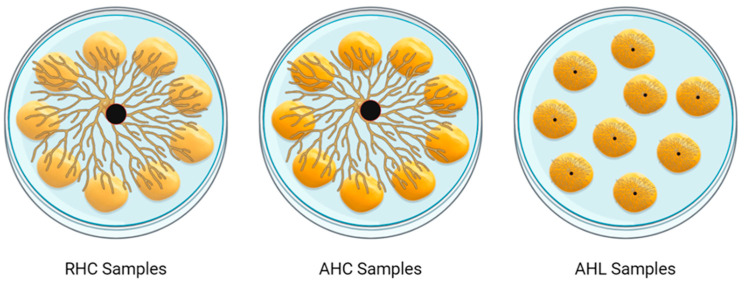
Graphic representation of inoculum techniques for lupin model system.

PHO-A and alkaloid production by *D. toxica* were evaluated on lupin beans in three different scenarios, summarized in Figure 5. First, rehydrated samples inoculated at the centre of a WA Petri dish (RHC) were prepared to simulate the best condition for PHO-A production by *D. toxica*, as previously described in paragraph 5.2.1. Dry lupins were sterilized at 121 °C for 15 min, then hydrated with sterile tap water in a 1:2 (*w*/*v*) ratio for 30 min under agitation until a_w_ equal to 0.98 ± 0.01 was reached. Successively, ten lupins were disposed of in a WA Petri dish encircling a round hyphal plug (ø 8 mm) picked up from a full-grown culture. Successively, two different procedures were used to inoculate commercial lupins, with a a_w_ of 1.00. In the first procedure, commercial lupins (without NaCl) were sterilized at 121 °C for 15 min and successively disposed of in a WA Petri dish encircling a round hyphal plug (ø 8 mm) picked up from a full-grown culture to simulate a hyphal cross-contamination after processing (AHC samples). In the second procedure, commercial lupins (without NaCl) were sterilized at 121 °C for 15 min, then an incision of 8 × 8 × 1 mm was made on the bean surface with a sterile scalpel. A round hyphal portion of 8 mm diameter was picked up from a full-grown culture and placed into the incision by a sterile pipette tip. Ten lupins were inoculated in an empty Petri dish with the above-reported procedure (AHL samples). A non-inoculated control sample was produced to evaluate whether the changes in PHO-A and alkaloid content could be attributed to other factors, not strictly related to fungal growth.

#### 5.3.2. UHPLC-MS/MS Quantification of PHO-A

PHO-A presence and quantification in contaminated lupin samples were assessed according to the method proposed by Eugelio et al. (2024), where further details on method validation are reported [32]. Briefly, samples were prepared as follows: 200 mg of lupin samples were mechanically reduced to a slurry, and then extracted with acetonitrile (ACN):H_2_O 80:20 (*v*/*v*) using Precellys Evolution homogenizer (Bertin Technologies, Montigny-Le-Bretonneux, France) at 3 cycles of 10 s, with a 30 s stop between each cycle at 7500 rpm. The obtained extracts were centrifuged at 11,424 rcf for 5 min, and 500 μL of supernatant was sequentially collected. The extraction solvent was removed using a SpeedVac Concentrator (Thermo Fisher Scientific, Waltham, MA, USA), and the precipitate was resuspended in 500 μL of H_2_O with 0.1% trifluoroacetic acid. Samples were filtered through Amicon Ultra-0.5 Centrifugal Fiter 3K Devices and centrifuged at 14,000 rcf for 30 min. A μ-SPE clean-up step was finally performed to remove any interference compound from the lupin matrix. After sample extraction, the PHO-A in samples was determined by an ACQUITY UPLC H-Class System from Waters Corporation (Milford, CT, USA) coupled with a 4500 QTrap mass spectrometer (Sciex, Toronto, ON, Canada), equipped with an Electrospray Ionization source (ESI). Tests were performed in triplicate.

#### 5.3.3. HPLC-MS/MS Quantification of QA

Lupin bean samples were processed according to Eugelio et al. (2023) [33]. In total, 200 mg of homogenised samples were extracted with 1 mL of a MeOH:H_2_O solution in a 60:40 (*v*/*v*) ratio by a Precellys Evolution homogenizer with 3 cycles of 10 s, at 7500 rpm, with a 45 s interval between each cycle. Then, samples were centrifuged at 4 °C at 11,424 rcf for 10 min. Then, 50 µL of supernatant was collected and diluted up to 1 mL, with a final H_2_O:MeOH ratio equal to 90:10 (*v*/*v*). The resulting solution was passed through to polymeric SPE cartridges as the clean-up step. The same LC-MS/MS equipment (Milford, CT, USA; Sciex, Toronto, ON, Canada) was used. Tests were performed in triplicate.

### 5.4. Data Analysis

Software Analyst 1.7.2 and Multiquant 3.0.3 (Sciex, Toronto, ON, Canada) were used for LC-MS/MS data collection and processing, and quantitative analysis, respectively, for the PHO-A and alkaloids. Statistical analysis of the data was carried out by using GraphPad Prism 8.0.2 (GraphPad Software, Inc.). Analysis of variance, followed by Tukey’s test, was used to determine significant differences in PHO-A or QA production among different samples.

## Data Availability

The datasets presented in this article are not readily available because the data are part of an ongoing study. Requests to access the datasets should be directed to the corresponding author.

## References

[1] Bennett J.W., Klich M. (2003). Mycotoxins. Clin. Microbol. Rev..

[2] Karlovsky P., Suman M., Berthiller F., De Meester J., Eisenbrand G., Perrin I., Oswald I.P., Speijers G., Chiodini A., Recker T. (2016). Impact of food processing and detoxification treatments on mycotoxin contamination. Mycotoxin Res..

[3] Okasha H., Song B., Song Z. (2024). Hidden hazards revealed: Mycotoxins and their masked forms in poultry. Toxins.

[4] Cowley R.B., Ash G.J., Harper J.D.I., Luckett D.J. (2012). Evaluation of resistance to Phomopsis stem blight (caused by *Diaporthe toxica*) in *Lupinus albus*. Eur. J. Plant Pathol..

[5] Van Warmelo K.T., Marasas W.F.O. (1972). *Phomopsis leptostromiformis*: The causal fungus of lupinosis, a mycotoxicosis, in sheep. Mycologia.

[6] Schloß S., Wedell I., Koch M., Rohn S., Maul R. (2015). Biosynthesis and characterization of ^15^N_6_-labeled phomopsin A, a lupin associated mycotoxin produced by *Diaporthe toxica*. Food Chem..

[7] Palmieri S., Eugelio F., Della Valle F., Fanti F., Buccioni F., Ricci A., Sergi M., Del Carlo M., Compagnone D. (2024). Molecularly imprinted polymer coupled to UHPLC-MS/MS for the analysis of phomopsins in lupin samples. Talanta.

[8] Williamson N.P., Highet A.S., Gams W., Sivasithamparam K., Cowling W.A. (1994). *Diaporthe toxica* sp. nov., the cause of lupinosis in sheep. Mycol. Res..

[9] Gresta F., Oteri M., Scordia D., Costale A., Armone R., Meineri G., Chiofalo B. (2023). White lupin (*Lupinus albus* L.), an alternative legume for animal feeding in the Mediterranean area. Agriculture.

[10] EFSA Panel on Contaminants in the Food Chain (CONTAM) (2012). Scientific opinion on the risks for animal and public health related to the presence of phomopsins in feed and food. EFSA J..

[11] Su Y.-Y., Qi Y.-L., Cai L. (2012). Induction of sporulation in plant pathogenic fungi. Mycology.

[12] Murali T.S., Suryanarayanan T.S., Geeta R. (2006). Endophytic *Phomopsis* species: Host range and implications for diversity estimates. Can. J. Microbiol..

[13] Nagarajan K., Tong W.Y., Leong C.R., Tan W.N. (2021). Potential of endophytic *Diaporthe* sp. as a new source of bioactive compounds. J. Microbiol. Biotechnol..

[14] Crowther T.W., Boddy L., Maynard D.S. (2018). The use of artificial media in fungal ecology. Fungal Ecol..

[15] Mattoo A.J., Nonzom S. (2022). Investigating diverse methods for inducing sporulation in endophytic fungi. Stud. Fungi.

[16] Książkiewicz M., Rychel-Bielska S., Plewiński P., Nuc M., Irzykowski W., Jędryczka M., Krajewski P. (2021). The resistance of narrow-leafed lupin to *Diaporthe toxica* is based on the rapid activation of defense response genes. Int. J. Mol. Sci..

[17] Udayanga D., Liu X., McKenzie E.H., Chukeatirote E., Bahkali A.H.A., Hyde K.D. (2011). The genus *Phomopsis*: Biology, application, species concepts and names of common phytopathogens. Fungal Divers..

[18] McR Wood P., Brown A.G.P. (1975). *Phomopsis*: The causal fungus of lupinosis. J. Dep. Agric. West. Aust..

[19] Shivas R.G., Allen J.G., Williamson P.M. (1991). Intraspecific variation demonstrated in *Phomopsis leptostromiformis* using cultural and biochemical techniques. Mycol. Res..

[20] Kunz B.M., Pförtner L., Weigel S., Rohn S., Lehmacher A., Maul R. (2022). Growth and toxin production of phomopsin A and ochratoxin A forming fungi under different conditions in a pea (*Pisum sativum*) model system. Mycotoxin Res..

[21] Zhang Y., Han T., Ming Q., Wu L., Rahman K., Qin L. (2012). Alkaloids produced by endophytic fungi: A review. Nat. Prod. Commun..

[22] Liu J.Y., Song Y.C., Zhang Z., Wang L., Guo Z.J., Zou W.X., Tan R.X. (2004). *Aspergillus fumigatus* CY018, an endophytic fungus in *Cynodos dactylon* as a versatile producer of new and bioactive metabolites. J. Biotechnol..

[23] Kaul S., Gupta S., Ahmed M., Dhar M.K. (2012). Endophytic fungi from medicinal plants: A treasure hunt for bioactive metabolites. Phytochem. Rev..

[24] Xu T.-C., Lu Y.-H., Wang J.-F., Song Z.-Q., Hou Y.-G., Liu S.-S., Liu C.-S., Wu S.-H. (2021). Bioactive secondary metabolites of the genus *Diaporthe* and anamorph *Phomopsis* from terrestrial and marine habitats and endophytes. Microorganisms.

[25] Rajput A., Sharma R., Bharti R. (2022). Pharmacological activities and toxicities of alkaloids on human health. Mater. Today Proc..

[26] Griffiths M.R., Strobel B.W., Hama J.R., Cedergreen N. (2021). Toxicity and risk of plant-produced alkaloids to *Daphnia magna*. Environ. Sci. Eur..

[27] Verma V.C., Kharwar R.N., Strobel G.A. (2009). Chemical and functional diversity of natural products from plant associated endophytic fungi. Nat. Prod. Commun..

[28] Helander M., Phillips T., Faeth S.H., Bush L.P., McCulley R., Saloniemi I., Saikkonen K. (2016). Alkaloid quantities in endophyte-infected tall fescue are affected by the plant-fungus combination and environment. J. Chem. Ecol..

[29] Gefrom A., Zeyner A., Georgiev V., Pavlov A. (2017). Reducing of alkaloid contents during the process of lactic acid silaging. Alkaloids—Alternatives in Synthesis, Modification and Application.

[30] Rathbone D.A., Lister D.L., Bruce N.C. (2002). Biotrasformation of alkaloids. Alkaloids Chem. Biol..

[31] Molina-Hernandez J.B., Laika J., Peralta-Ruiz Y., Palivala V.K., Tappi S., Cappelli F., Ricci A., Neri L., Chaves-López C. (2022). Influence of atmospheric cold plasma exposure on naturally present fungal spores and physicochemical characteristics of sundried tomatoes (*Solanum lycopersicum* L.). Foods.

[32] Eugelio F., Palmieri S., Fanti F., Buccioni F., Oliva E., Paparella A., Del Carlo M., Compagnone D., Sergi M. (2024). Development and validation of analytical method by micro-solid-phase extraction followed by ultra high performance liquid chromatography coupled to tandem mass spectrometry analysis for the quantification of Phomopsin A in lupin samples. J. Chromatogr. Open.

[33] Eugelio F., Palmieri S., Fanti F., Messuri L., Pepe A., Compagnone D., Sergi M. (2023). Development of an HPLC-MS/MS method for the determination of alkaloids in lupins. Molecules.

